# Based on Integrated Bioinformatics Analysis Identification of Biomarkers in Hepatocellular Carcinoma Patients from Different Regions

**DOI:** 10.1155/2019/1742341

**Published:** 2019-10-28

**Authors:** Linxin Teng, Kaiyuan Wang, Yu Liu, Yanxia Ma, Weiping Chen, Lei Bi

**Affiliations:** School of Preclinical Medicine, Nanjing University of Chinese Medicine, 138 Xianlin Road, Nanjing, Jiangsu 210023, China

## Abstract

Accumulating statistics have shown that liver cancer causes the second highest mortality rate of cancer-related deaths worldwide, of which 80% is hepatocellular carcinoma (HCC). Given the underlying molecular mechanism of HCC pathology is not fully understood yet, identification of reliable predictive biomarkers is more applicable to improve patients' outcomes. The results of principal component analysis (PCA) showed that the grouped data from 1557 samples in Gene Expression Omnibus (GEO) came from different populations, and the mean tumor purity of tumor tissues was 0.765 through the estimate package in R software. After integrating the differentially expressed genes (DEGs), we finally got 266 genes. Then, the protein-protein interaction (PPI) network was established based on these DEGs, which contained 240 nodes and 1747 edges. FOXM1 was the core gene in module 1 and highly associated with FOXM1 transcription factor network pathway, while FTCD was the core gene in module 2 and was enriched in the metabolism of amino acids and derivatives. The expression levels of hub genes were in line with The Cancer Genome Atlas (TCGA) database. Meanwhile, there were certain correlations among the top ten genes in the up- and downregulated DEGs. Finally, Kaplan–Meier curves and receiver operating characteristic (ROC) curves were plotted for the top five genes in PPI. Apart from CDKN3, the others were closely concerned with overall survival. In this study, we detected the potential biomarkers and their involved biological processes, which would provide a new train of thought for clinical diagnosis and treatment.

## 1. Introduction

Liver cancer is highly fatal, which causes the second highest death rate of cancer-related mortality worldwide [1,2]. Globally, it is estimated that approximately 80% of liver cancers were HCC [3]. Nobody disputes that this is a public health challenge that needs widespread attention. HCC is a multigene disease caused by the interaction of multiple cancer-promoting and suppressing genes with the microenvironment, and its molecular mechanism is still unclear. Thus, the identification of new potential therapeutic targets is urgently needed.

In recent years, despite the advances in our knowledge of the genetic factors, it is a pity that the death rates were increasing rapidly [4, 5]. If the HCC patients can be diagnosed early, the survival rate may be greatly improved by means of liver resection [6, 7]. However, due to the late diagnosis of most patients with HCC, the physical condition is not good enough to withstand the risk of surgery [8, 9]. What is worse, the survival rate of patients with advanced HCC is further decreased due to the widespread resistance to chemotherapy. Sorafenib, for example, a multikinase inhibitor, is widely used for the treatment of patients with advanced HCC with a long application time [10, 11], but patients invariably develop sorafenib resistance and it only provides limited survival benefit [12]. As a result, we are badly in need of finding new diagnostic and prognostic markers for HCC, which might facilitate early diagnosis and guide treatment decisions to improve patients' survival and quality of life.

In this study, we downloaded the expression matrix of six datasets from the Gene Expression Omnibus (GEO) database, including 630 adjacent normal and 927 tumor tissues. PCA, tumor purity evaluation, and differential expression gene (DEG) analysis were performed by using R software. 266 DEGs were finally obtained, consisting of 81 upregulated and 185 downregulated genes. FunRich undertook the entire enrichment analyses in our experiment, while Cytoscape was employed to build a network diagram. We had found that upregulated genes were closely related to mitotic cell cycle. Different from upregulated genes, downregulated genes were enriched in lipid and lipoprotein derivative pathway. To further explore the role of these DEGs, we divided the PPI network into several independent modules. FOXM1 and FTCD were core genes in two separate models with the highest score, respectively. The former is enriched in FOXM1 transcription factor network pathway, while the latter is mainly enriched in the metabolism of amino acid and derivative pathway. Finally, The Cancer Genome Atlas (TCGA) data were used to test our results and predict overall survival related to five hub genes in PPI. We found certain correlations among hub genes, which might reveal potential signaling pathways in HCC. And 4 of 5 hub genes were connected with low overall survival of HCC patients. Undoubtedly, recognition of biomarkers in HCC that plays a key role in disease progression can provide new insights into the development, prognosis, and treatment of HCC.

## 2. Materials and Methods

### 2.1. Tumor Purity Estimation and Differential Expression Gene Analysis

All the gene expression profile data originated from the GEO database (https://www.ncbi.nlm.nih.gov/geo). There were huge liver cancer mRNA microarray datasets in the GEO database, and the included datasets need to meet the following conditions: (1) the microarray data were available; (2) they contained at least 100 samples; and (3) they employed tumor and adjacent normal tissues. Therefore, we selected the following datasets: GSE25097, GSE36376, GSE45436, GSE54236, GSE64041, and GSE112790. GEOquery package in R/Bioconductor software (version 3.6.1, https://www.r-project.org) was used to get datasets, which was applied to download gene expression and probe annotation information for the selected datasets. Then, the estimate package was used to estimate tumor purity, while the limma package for data normalization and gene differential expression matrix acquisition. In the differential expression gene analysis, FDR < 0.05 and |log_2_FC| ≥ 1 were considered to be significant DEGs, which were visualized by ggplot2 package.

### 2.2. Enrichment Analysis

We divided the DEGs into two categories and ranked them in descending order of absolute values. Since the six datasets were not from the same platform, we used RRA package to integrate the DEGs. FunRich (version 3.1.3, http://www.funrich.org) is such powerful stand-alone software that we primarily used to perform functional enrichment analysis [13]. Biological process, biological pathway, cellular component, and molecular function can be achieved by FunRich in the present study.

### 2.3. PPI Network and Module Analysis

The Search Tool for the Retrieval of Interacting Genes database (STRING, https://string-db.org) can provide information on protein interactions, whose data mainly came from structural predictions and literature reports [14]. Combined score ≥ 0.4 was considered as the cutoff value, and the filtered node information was saved locally for subsequent visualization. Then, we used Cytoscape software (version 3.6.0, https://cytoscape.org) to build the protein-protein interaction (PPI) network and one of the plug-in in Cytoscape named Molecular Complex Detection (MCODE) was applied to detect notable modules in this PPI network [15]. As is known to all, network modules, as one of the characteristics of protein networks, may have specific biological significance. The default advanced option parameters (degree cutoff = 2, node score cutoff = 0.2, and k-core = 2) in MCODE already met our requirements, so we did not modify it. Moreover, models with score ≥ 5 were used for further path enrichment analysis, which can help to explore the potential biological functions of DEGs.

### 2.4. Analysis for Expression Level and Correlation of the Hub Genes

The Gene Expression Profiling Interactive Analysis (GEPIA, http://gepia.cancer-pku.cn) is an online website tool that can perform analysis including gene expression analysis and correlation analysis [16]. Data from TCGA and the Genotype-Tissue Expression (GTEx, http://commonfund.nih.gov/GTEx/) were used to apply a standard processing pipeline before being used by GEPIA. Based on the huge amount of data from GEPIA, we used it to demonstrate the expression of hub genes in LIHC tissues and normal ones and then made a boxplot to visualize the results. There are three correlation coefficients (Pearson, Spearman, and Kendall) for users to choose in GEPIA, and any sets given by TCGA and/or GTEx expression data were used to check the relative ratios between two genes.

### 2.5. Overall Survival Analysis and ROC Curve Analysis of Hub Genes

Kaplan–Meier plotter (KM plotter, http://kmplot.com/analysis/) is a database that can be accessed openly, which is the largest dataset including breast, ovarian, lung, and gastric cancer [17]. This database is rich in gene expression data and overall survival information from TCGA, which we can use to draw survival curves with 95% confidence interval hazard ratio and log-rank *P* value. Receiver operating characteristic (ROC) curve analysis was employed to verify the diagnostic performance of hub genes, and 3 years was set as the predicted time. Multivariate Cox proportional hazards regression analysis was performed based on hub genes. The risk score for predicting overall survival was calculated as follows: risk  score = ∑_*i*=1_^*n*^(coef_*i*_ *∗* Expr_*i*_), where coef is the regression coefficient and Expr is the expression level of the gene. Then, according to the mean risk score, samples were divided into low- and high-risk groups. Finally, survival analysis and ROC curve analysis of the risk score were performed by using the same method as described above.

## 3. Results

### 3.1. Tumor Purity Estimation of Tumor Tissue in Datasets and Identification of DEGs

**Figure 1:**
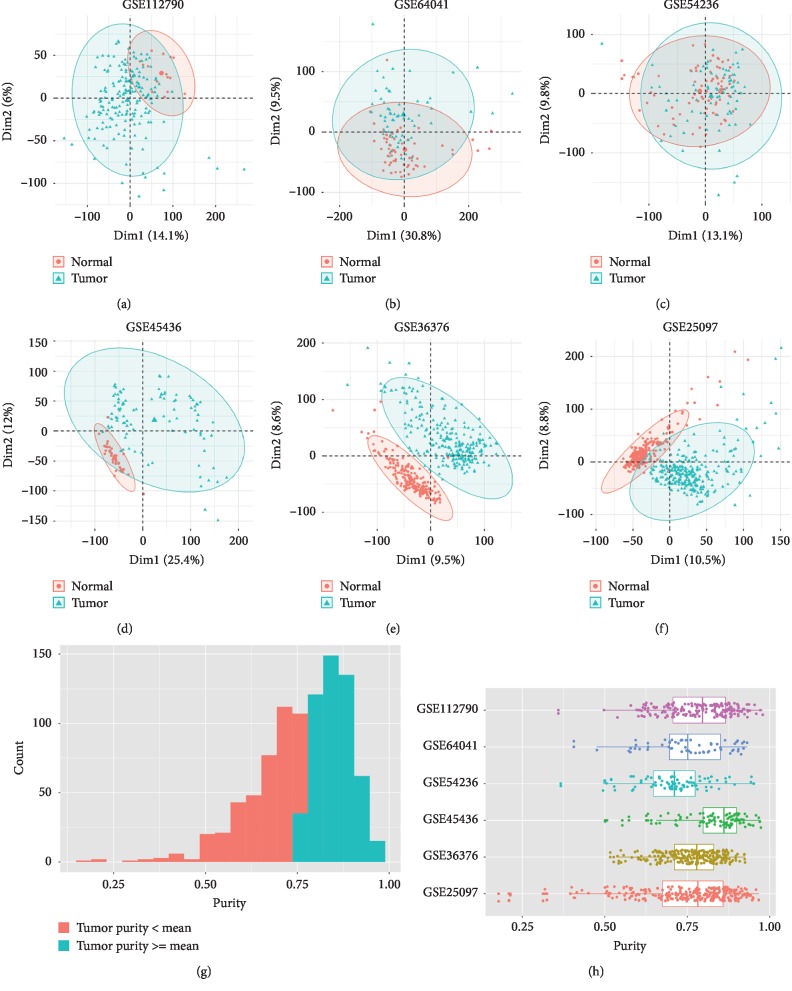
PCA of datasets and visual representation of tumor purity estimation. PCA plot of (a) GSE112790, (b) GSE64041, (c) GSE54236, (d) GSE45436, (e) GSE36376, and (f) GSE25097. (g) Histogram of tumor purity of all tumor tissues. (h) Boxplot of tumor purity in each dataset.

**Figure 2:**
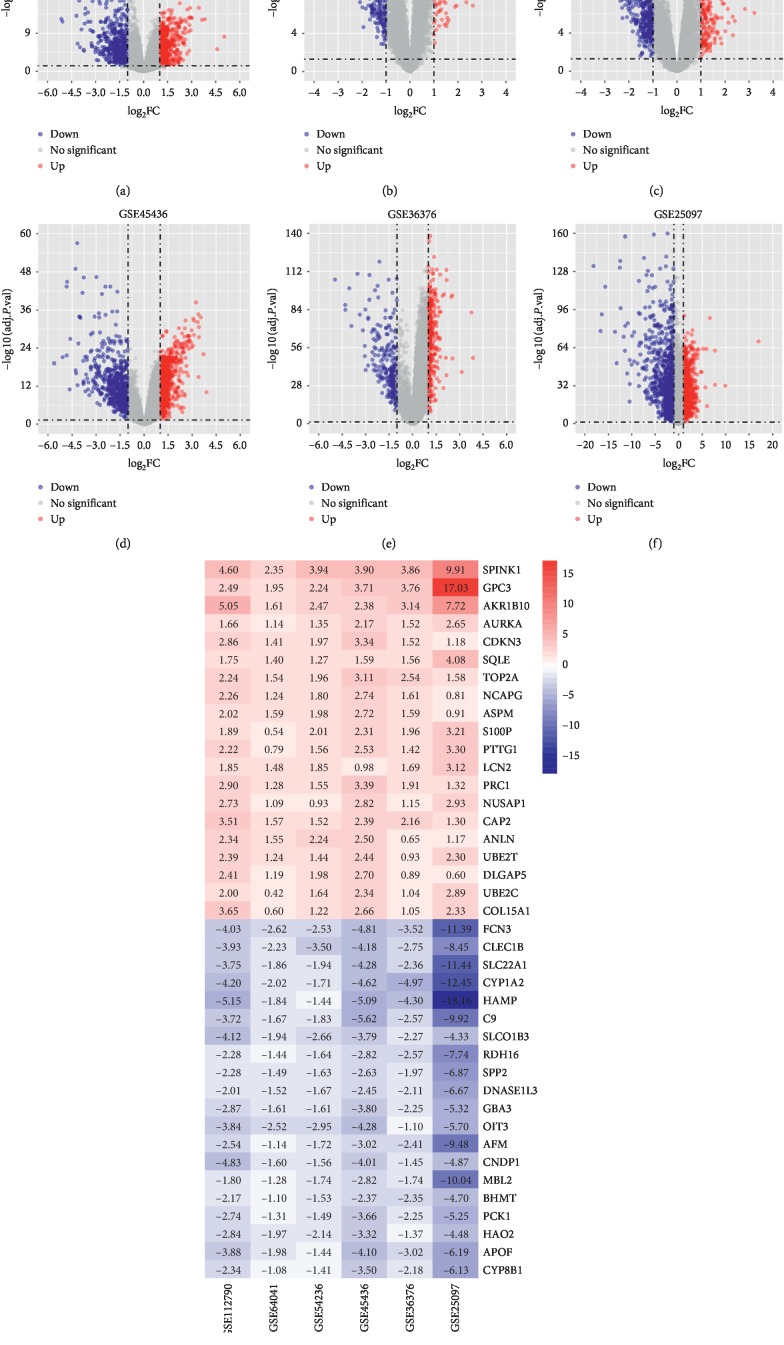
Volcano plot of DEGs and heatmap of the top 10 up- and downregulated genes. Volcano plot of (a) GSE112790, (b) GSE64041, (c) GSE54236, (d) GSE45436, (e) GSE36376, and (f) GSE25097. Red and blue dots in the volcano plot represent upregulated and downregulated genes, respectively, while gray represents genes that have no significant difference. (g) Heatmap of the top 10 upregulated and downregulated genes. The number in each rectangle represents the gene expression level.

630 normal and 927 tumor samples were selected in this study (Table 1). The results of PCA showed that the normal control group and the tumor group in the six datasets could be discriminated very well (Figures 1(a)–1(f)). Then, we calculated the tumor purity of 927 liver tumor tissues through the estimate algorithm. As shown in Figures 1(g) and 1(h), the purity of tumors ranged from 0.179 to 0.979 and 55.8% of the tumor samples had a greater value than the mean value of 0.765. After performing differential expression gene analysis on each dataset, 81 upregulated and 185 downregulated genes were finally detected by RRA (Supplementary [Supplementary-material supplementary-material-1]). Compared with the adjacent normal ones, the expression of these genes in tumor tissues was all upregulated or downregulated ≥ 2-folds (Figures 2(a)–2(f)). We sorted the upregulated and downregulated genes in ascending order according to the FDR values and created the heatmap with the top 20 genes (Figure 2(g)).

### 3.2. Enrichment Analysis of DEGs

**Figure 3:**
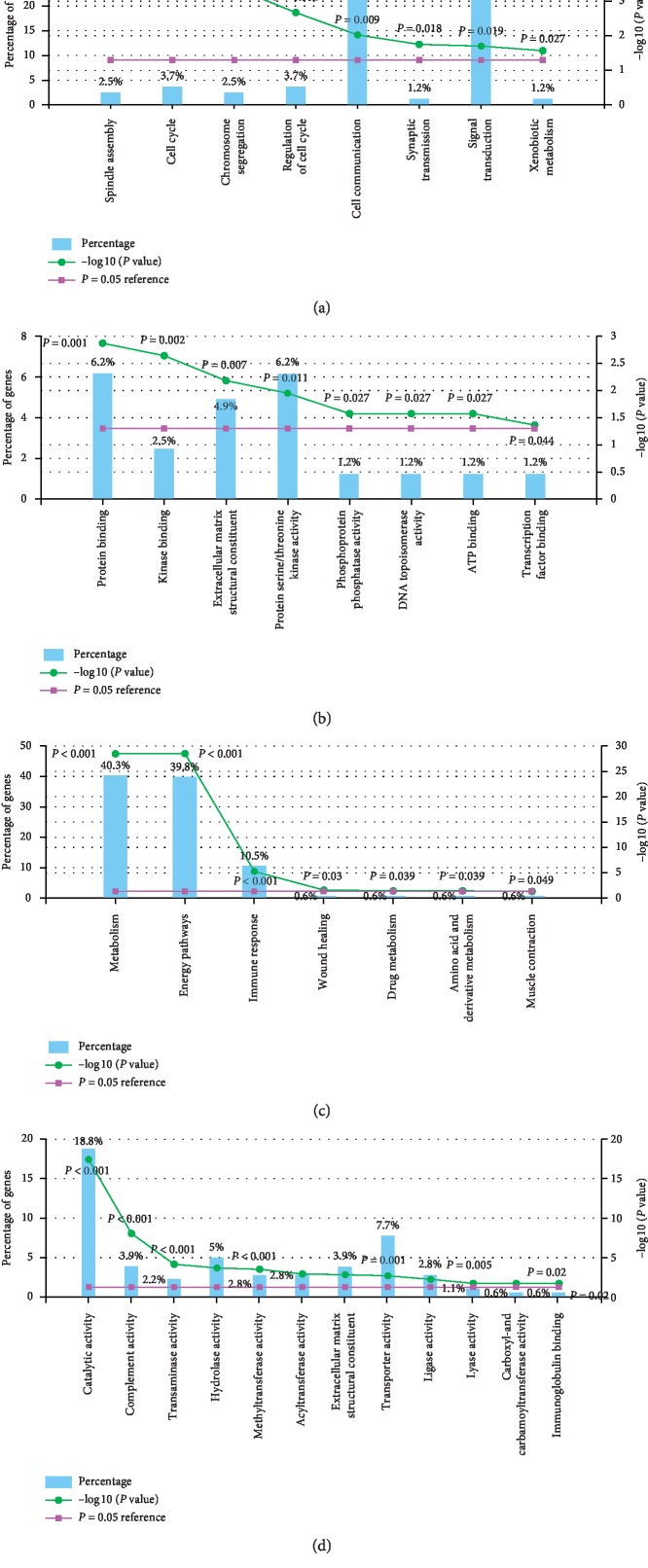
Histograms of molecular function and biological process for up- and downregulated genes. (a) Biological process for upregulated genes. (b) Molecular function for upregulated genes. (c) Biological process for downregulated genes. (d) Molecular function for downregulated genes.

**Figure 4:**
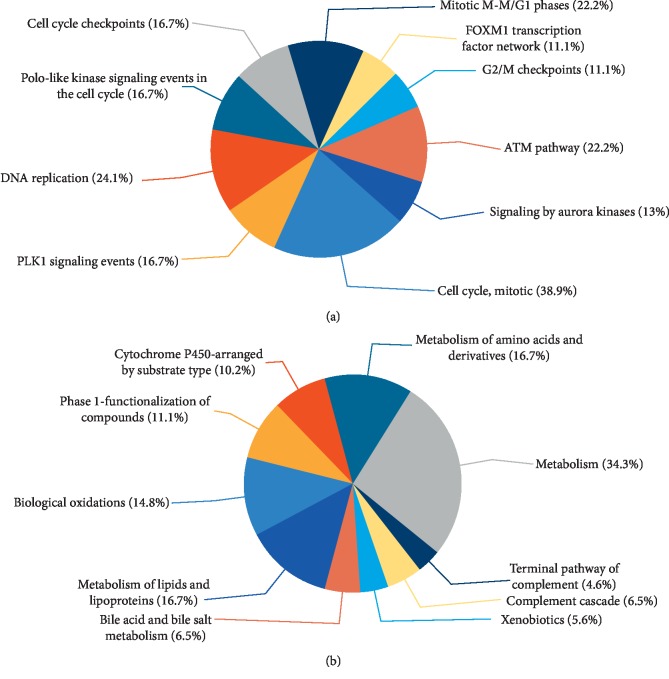
Pie charts of biological pathway for (a) upregulated genes and (b) downregulated genes.

DEGs from six independent datasets were integrated and introduced into FunRich for enrichment analysis. The biological processes for upregulated genes were mainly associated with spindle assembly and cell cycle (Figure 3(a) and Supplementary [Supplementary-material supplementary-material-1]), while the molecular functions were about protein binding and kinase binding (Figure 3(b) and Supplementary [Supplementary-material supplementary-material-1]). In addition, the vital gene of upregulated genes was called FOXM1, which was highly correlated with transcription factor activity and FOXM1 transcription factor network (Supplementary [Supplementary-material supplementary-material-1]). The functional enrichment of downregulated genes was associated with metabolism, catalytic activity, and energy pathways (Figures 3(c) and 3(d)). And the pivotal gene FTCD in this group was enriched in methyltransferase activity, energy pathways, and histidine catabolism. Through the biological pathway enrichment analysis, we found that upregulated genes were closely related to mitotic cell cycle, DNA replication, mitotic G1-G1/S phases, and ATM pathway (Figure 4(a) and Supplementary [Supplementary-material supplementary-material-1]). Different from upregulated genes, downregulated genes were enriched in lipid and lipoprotein derivative pathway (Figure 4(b) and Supplementary [Supplementary-material supplementary-material-1]).

### 3.3. PPI Network Establishment and Pathway Analysis of Network Module

**Figure 5:**
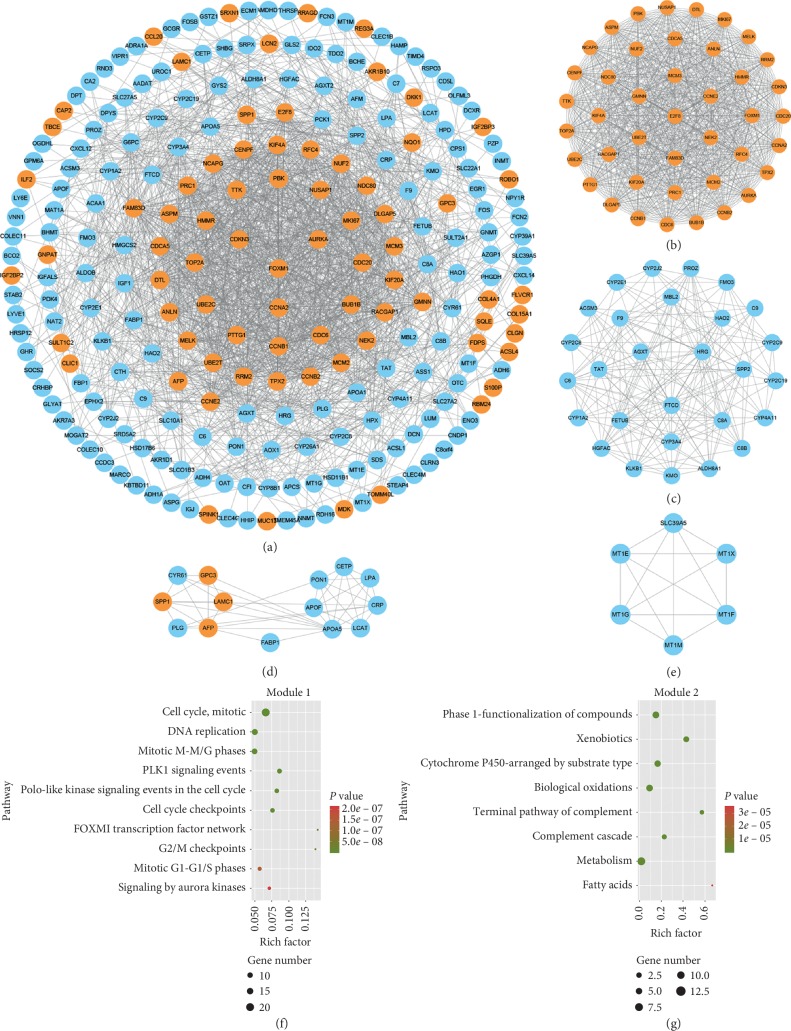
PPI network diagrams of DEGs and module network and pathway enrichment analysis of modules. (a) PPI network of DEGs. Red nodes represent upregulated genes and blue nodes represent downregulated genes. (b) Module 1, MCODE score = 40.78; (c) module 2, MCODE score = 11.778; (d) module 3, MCODE score = 6.615; (e) module 4, MCODE score = 5.6. Pathway analysis of (f) module 1 and (g) module 2.

After introducing the gene list into STRING website, we finally got the information of 240 nodes and 1747 edges (combined score ≥ 0.4). Then, the network diagram was presented by Cytoscape based on the STRING database (Figure 5(a) and Supplementary [Supplementary-material supplementary-material-1]). Interestingly, most of the nodes with higher connectivity were upregulated genes, which signified that they would be closer to the center of the circle. Four modules with score ≥ 5 were detected via MCODE (Figures 5(b)–5(e)). It can be seen in Figure 5(b) that the hub nodes were FOXM1, CCNA2, AURKA, CDKN3, and CDC20 in module 2. Besides, as shown in Figure 5(c), FTCD, HRG, AGXT, C8A, and TAT were nodes with highest connectivity in module 2. Among the four models, only model 3 contained both of the protein nodes expressed by up- and downregulated genes, including AFP, PLG, CRP, FABP1, and SPP1. And the results of the pathway analysis for the two modules with the highest combined score are shown in Figures 5(f) and 5(g) and Supplementary [Supplementary-material supplementary-material-1]. It is worth mentioning the most significant pathways in module 1 and module 2 were mitotic cell cycle and phase 1—functionalization of compounds, respectively.

### 3.4. Expression Level and Correlation of Hub Genes

**Figure 6:**
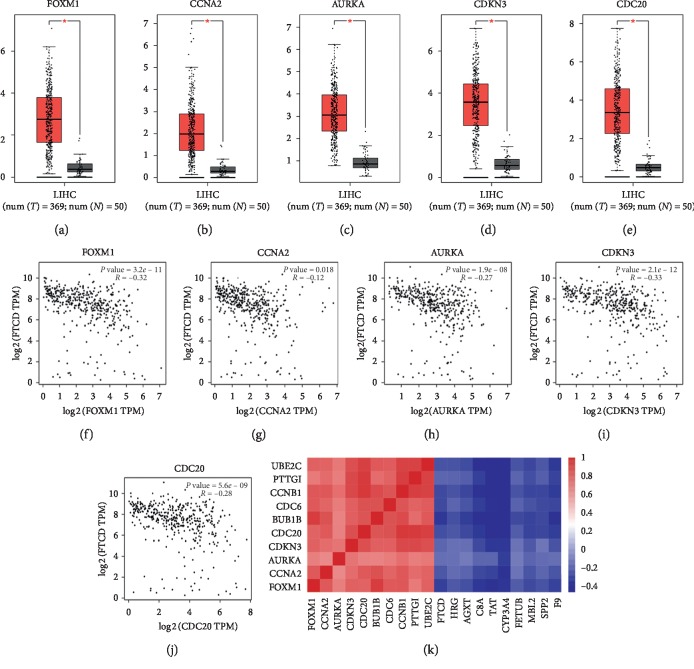
Top five hub genes expression level in liver hepatocellular carcinoma (LIHC) and correlation analysis among hub genes. (a–e) Boxplots of expression level of FOXM1, CCNA2, AURKA, CDKN3, and CDC20. (f–j) Scatter plots of correlation analysis of FTCD and the top five hub genes. (k) Heatmap of correlation coefficients among the top ten hub genes in module 1 and module 2.

A total of 419 samples were selected for gene expression level analysis, including 369 tumor tissues and 50 normal liver tissues. As shown in Figures 6(a)–6(e), the expression of the five hub genes in cancer tissues was significantly higher than that in normal ones. Moreover, it turned out by the correlation analysis that the increased expression of these genes was strongly correlated with the decreased expression of FTCD (Figures 6(f)–6(j)), and heatmap of correlation coefficients between hub genes was shown in Figure 6(k).

### 3.5. Prognostic Factor Detection and Validation

**Figure 7:**
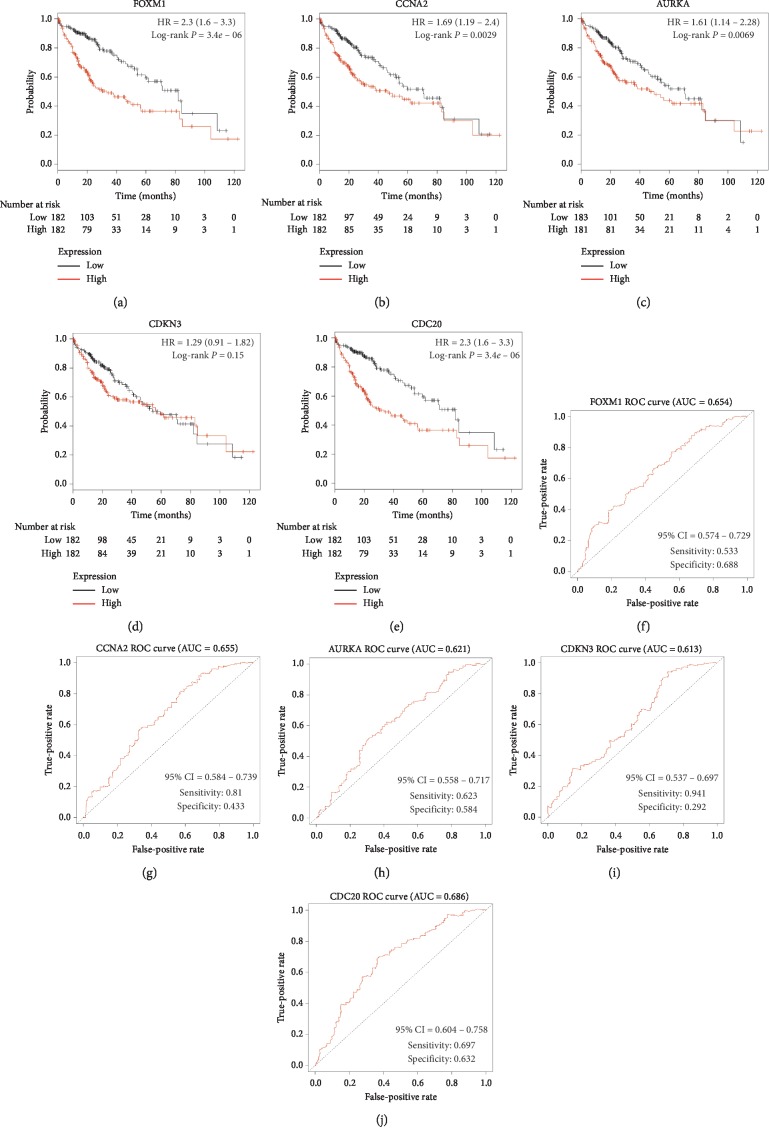
Overall survival analysis and ROC curve analysis of the top five hub genes. (a–e) Kaplan–Meier curves for FOXM1, CCNA2, AURKA, CDKN3, and CDC20. (f–j) ROC curves for predicting 3-year survival in HCC patients based on FOXM1, CCNA2, AURKA, CDKN3, and CDC20.

**Figure 8:**
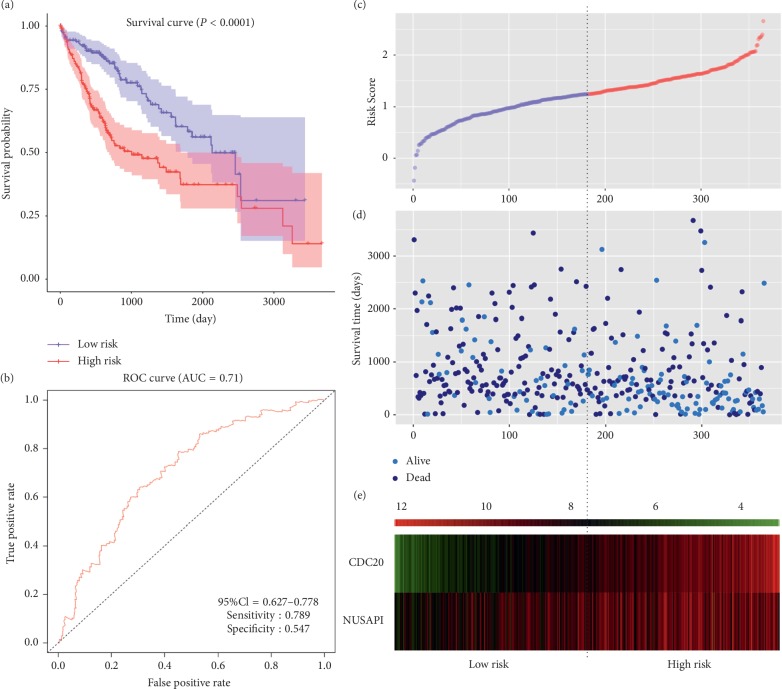
Prognostic risk score model in HCC patients. (a) Kaplan–Meier curve for low- and high-risk groups. (b) ROC curve for predicting survival in HCC patients based on the risk score. (c) Volcano plot of the risk score of each individual. (d) Volcano plot of survival status and survival time of each individual. (e) Heatmap of CDC20 and NUSAP1 in low- and high-risk groups.

The analysis of 364 HCC patients clearly showed that 4 out of 5 genes with the highest degree in PPI had a significant impact on prognosis (Figure 7). High expression of FOXM1 (HR = 1.68; *P*=0.0033), CCNA2 (HR = 1.69; *P*=0.0029), AUPKA (HR = 1.61; *P*=0.0069), CDKN3 (HR = 1.29; *P*=0.15), and CDC20 (HR = 2.3; *P*=3.4*e* − 06) indicated worse survival rate in patients with HCC. In ROC curve analysis, FOXM1 (AUC = 0.654, sensitivity = 0.533, and specificity = 0.688), CCNA2 (AUC = 0.655, sensitivity = 0.81, and specificity = 0.433), AUPKA (AUC = 0.621, sensitivity = 0.623, and specificity = 0.584), CDKN3 (AUC = 0.613, sensitivity = 0.941, and specificity = 0.292), and CDC20 (AUC = 0.686, sensitivity = 0.697, and specificity = 0.632) had certain predictive value for the 3-year survival rate of HCC patients. All of the top 20 genes in PPI exhibited a significant prognostic value (Supplementary [Supplementary-material supplementary-material-1]). Then, risk score model was built based on CDC20 (coef = 0.349; *P*=2.8*e* − 07) and another gene among the top 20 genes, named NUSAP1 (coef = −0.182; *P*=0.046). Compared with overall survival prediction with a single gene, the risk score model had a higher predictive value (AUC = 0.71, sensitivity = 0.789, and specificity = 0.547) (Figure 8).

## 4. Discussion

In the present study, we had detected totally 266 DEGs. FOXM1 was the most connected gene in upregulated genes in the PPI network, which had 44 edges. Increasing evidence has suggested that FOXM1 is elevated in many tumors, such as intrahepatic cholangiocarcinoma, oesophageal adenocarcinoma, gastric cancer, cervical cancer, and HCC [18–24]. Since FOXM1 can promote the proliferation and invasion of cancer cells, it may give rise to the poor prognosis and low survival rate of patients with high FOXM1 expression [22, 24–31]. Not only that, it was also found that FOXM1 contributes to tumor angiogenesis in the study of colorectal and gastric cancer [23, 32]. In previous studies, there was a large amount of evidence that FOXM1 directly or indirectly affects the occurrence and development of HCC, which is in line with our results [27–29, 33–39]. In addition, in an in vivo study of HCC, the growth of tumors in mice with FOXM1 deficiency was completely stagnated, suggesting that FOXM1 has the potential to become an independent biomarker of HCC [31]. It is worth mentioning that KIF4A has been confirmed to be a downstream target of FOMX1 and the expression level of KIF4A is positively correlated with FOXM1. Overexpression of both genes will lead to excessive cell proliferation and promote tumor development [22]. Meanwhile, we observed a significant increase in the expression of KIF4A in our experimental results (*P*=3.18*e* − 07), which coincided with previous studies [22]. MircoRNA plays an active role in HCC as well. For instance, the expression of microRNA-135a transcribed by FOXM1 can affect the prognosis and survival rate of patients with HCC [40]. Unfortunately, we did not build a competing endogenous RNAs (ceRNA) network in this project to find potential downstream noncoding RNAs for FOXM1, which should be investigated in future study.

FTCD was the core gene in model 2 and had a certain correlation with FOXM1. Additionally, FTCD was found useful to distinguish early HCC from benign tumors, suggesting that it might be a potential marker for HCC early diagnosis [41]. The results of enrichment analysis for FTCD were consistent with prior reports that the decrease in FTCD expression impeded the degradation pathway of histidine, which leads to the poor performance of methotrexate [42]. Therefore, we infer that patients with HCC who were not responding to methotrexate may be associated with abnormal expression of FTCD. Besides, in the available evidence, we found that autoimmune hepatitis has a 0.6% to 0.7% probability of inducing HCC [43, 44]. Interestingly, by reducing the number of circulating autoreactive T cells, the increased expression of FTCD can prevent the progression of autoimmune hepatitis [45]. Thus, low level of FTCD might contribute to high incidence of HCC and serves as a useful biomarker for primary HCC. Further research will be needed to clarify the role of FTCD in tumorigenesis.

In addition to FOXM1, CCNA2, AURKA, CDKN3, and CDC20 can be seen in the forefront of PPI. It is common knowledge that CCNA2, a core cell cycle regulator, plays a critical role with high expression from S phase to early mitosis [46, 47]. It was reported that high expression of CCNA2 might induce hepatocyte nodular proliferation [48]. And the exosome circRNAs, secreted from liver adipocytes, promoted tumor growth by controlling miR-34a level and activating the USP7/CCNA2 signaling pathway [49]. All of the above findings indicate that CCNA2 directly or indirectly influences the development of HCC, which is considered as a vital part in HCC development. High expression of AURKA has previously been detected in different cancer types as well [50–52], which is implicated with the regulation of cell cycle and division [53]. With no exception to HCC, it is also described as an oncoprotein and therapeutic target. Microarray analysis pointed out that AURKA phosphorylated and stabilized hepatoma upregulated protein [54]. Moreover, a research revealed that AURKA can, in turn, give rise to malignant phenotypes of HCC by regulating HIF-1*α* through activation of AKT and p38-MAPK signaling pathways [55]. As a result, we conjecture that it may function as a cancer-promoting gene. Excessive replication of the centrosome is considered to be a common feature of almost all human cancers [56], and CDKN3 happens to have the ability to prevent this abnormality [57]. Further research indicated CDKN3 seemed to play a role in tumor suppression by CDC2 signaling pathway [58]. However, bioinformatics analysis for identification of molecular target genes in HCC revealed that the relative expression levels of CDKN3 were significantly upregulated in tumor tissues, which proved that our results were not accidental [59]. Therefore, we conjecture that the result may be due to the positive feedback regulation in the tumor microenvironment, which surely requires subsequent experiments to verify. Regrettably, since the expression level of CDKN3 is not associated with the prognosis of patients with HCC in our study, CDKN3 cannot be counted as a candidate biomarker accordingly. CDC20 played a pivotal role in the regulation of chromosome segregation and the timely end of mitosis [60]. Abnormal CDC20 expression has been detected in most human cancers [61–63], and CDC20 knockdown caused mitotic arrest to efficiently kill slippage-prone and apoptosis-resistant cancer cells [64], supporting an oncogenic role of CDC20. In conclusion, combined with literature reports and our findings, FOXM1, FTCD, CCNA2, AURKA, and CDC20 are very competitive biomarkers of HCC, while whether CDKN3 can be regarded as a biomarker for HCC remains further studies.

Prior to our research, there have been some reports on bioinformatics analysis of critical genes in HCC [65–68]. Nevertheless, our research still has several obvious advantages: firstly, the datasets we selected contain as many as 1557 samples and cover multiple different regions; secondly, we prioritized PCA of these datasets to ensure that tumor and normal tissues come from two distinct populations; thirdly, the method of tumor purity estimation allowed us to show readers the quality of the tumor samples in this study; fourthly, we have established a multivariate Cox proportional hazards regression model based on hub genes to improve the accuracy of single prognostic factor prediction; finally, we performed a correlation analysis between the upregulated and downregulated genes, which may reveal potential signal transduction pathways in HCC. We have to admit that our research still has the following shortcomings: first of all, our results were only based on GEO and TCGA data analysis and have not been verified; next in importance, there may be distinctions in gene expression among different types of tumors, which will be perfected in future experiments.

## 5. Conclusion

In conclusion, the integrated bioinformatics analysis was derived from 1557 tumor tissues and adjacent normal tissues in the GEO database. Tumor and normal samples came from different populations and half of the tumor samples have a purity of more than 0.765. 266 genes were eventually identified as candidate HCC biomarkers, which were enriched in signaling pathways closely related to cell proliferation and metabolic function. FOXM1, CCNA2, AURKA, CDKN3, and CDC20 were at the core of these genes, which opened up new horizons for diagnosis, prognosis, and treatment of HCC patients.

## Figures and Tables

**Table 1:** The gene expression profile data characteristics.

Record	Organism	Tissue	Normal (*n* = 630)	Tumor (*n* = 927)	Region	Platform
GSE25097	Homo sapiens	Liver tumor	243 (38.6%)	268 (28.9%)	Boston	GPL10687—Rosetta/Merck Human RSTA Affymetrix 1.0 microarray, custom CDF
GSE36376	Homo sapiens	Liver tumor	193 (30.6%)	240 (25.9%)	Seoul	GPL10558—Illumina HumanHT-12 V4.0 expression beadchip
GSE45436	Homo sapiens	Liver tumor	39 (6.2%)	95 (10.2%)	Taipei	GPL570—[HG-U133_Plus_2] Affymetrix Human Genome U133 Plus 2.0 Array
GSE54236	Homo sapiens	Liver tumor	80 (12.7%)	81 (8.7%)	Modena	GPL6480—Agilent-014850 Whole Human Genome Microarray 4x44K G4112F (Probe Name version)
GSE64041	Homo sapiens	Liver tumor	60 (9.5%)	60 (6.5%)	Basel	GPL6244—[HuGene-1_0-st] Affymetrix Human Gene 1.0 ST Array [transcript (gene) version]
GSE112790	Homo sapiens	Liver tumor	15 (2.4%)	183 (19.7%)	Tokyo	GPL570—[HG-U133_Plus_2] Affymetrix Human Genome U133 Plus 2.0 Array

## Data Availability

The data used to support the findings of this study are available from the corresponding author upon request.

## Supplementary Materials

Supplementary Materials Supplementary Table S1: information for the DEGs. Supplementary Table S2: enrichment analysis of the DEGs. Supplementary Table S3: node information for PPI network. Supplementary Table S4: pathway analysis of network modules. Supplementary Table S5: univariate Cox regression analysis of the prognosis of the top 20 genes in PPI.
